# Analyse zur Häufigkeit einer gerinnungshemmenden Medikation bei Patient*inn*en mit kognitiven Störungen und zerebraler Amyloidangiopathie (CAA)

**DOI:** 10.1007/s00115-023-01547-8

**Published:** 2023-09-25

**Authors:** R. Haußmann, P. Homeyer, M. Haußmann, C. Sauer, J. Linn, M. Donix, M. Brandt, V. Puetz

**Affiliations:** 1 Klinik und Poliklinik für Psychiatrie und Psychotherapie, Universitätsklinikum Carl Gustav Carus an der Technischen Universität Dresden, Dresden, Deutschland; 2 Dialysepraxis Leipzig, MVZ, Leipzig, Deutschland; 3 Institut und Poliklinik für diagnostische und interventionelle Neuroradiologie, Universitätsklinikum Carl Gustav Carus an der Technischen Universität Dresden, Dresden, Deutschland; 4 Klinik und Poliklinik für Neurologie, Universitätsklinikum Carl Gustav Carus an der Technischen Universität Dresden, Dresden, Deutschland; 5 DZNE, Deutsches Zentrum für Neurodegenerative Erkrankungen, Dresden, Deutschland; 6 Universitäts DemenzCentrum (UDC), Klinik und Poliklinik für Psychiatrie und Psychotherapie, Uniklinikum Dresden, Dresden, Deutschland; 7 Dresdner Neurovaskuläres Centrum (DNVC), Universitätsklinikum Carl Gustav Carus an der Technischen Universität Dresden, Dresden, Deutschland; 8 Klinik und Poliklinik für Psychiatrie und Psychotherapie, Uniklinikum Dresden, Dresden, Deutschland

**Keywords:** Thrombozytenaggregationshemmung, Orale Antikoagulation, Intrazerebrale Blutung, Kortikale Mikroblutung, Superfizielle kortikale Siderose, Platelet aggregation inhibition, Oral anticoagulation, Intracerebral bleeding, Cortical microbleeding, Superficial cortical siderosis

## Abstract

**Ziel der Arbeit:**

Analyse der Häufigkeit einer zerebralen Amyloidangiopathie (CAA) bei Patient*inn*en mit kognitiven Störungen und der Häufigkeit einer Koinzidenz von gerinnungshemmender Therapie und CAA. Explorative Analyse von Zusammenhängen zwischen Antikoagulation und CAA-typischer Läsionslast in der MRT.

**Material und Methoden:**

Patient*inn*en mit subjektiver kognitiver Störung (SCD), amnestischem und nichtamnestischem MCI (aMCI/naMCI), Alzheimer-Demenz (AD), gemischter Demenz (MD) und vaskulärer Demenz (VD) aus einem universitären Demenzzentrum, die sich von 02/2016 bis 12/2020 erstmals zur Demenzdiagnostik vorgestellt hatten, wurden in diese retrospektive Analyse eingeschlossen. Im Rahmen der Diagnostik erfolgten eine kranielle MRT inkl. Gradientenechosequenz und die Erfassung CAA-spezifischer MRT-Biomarker. Im Rahmen der retrospektiven Aktendurchsicht wurde das Vorliegen einer gerinnungshemmenden Medikation mit Thrombozytenaggregationshemmern, direkten oralen Antikoagulanzien (DOAK) oder Vitamin-K-Antagonisten zum Zeitpunkt der Vorstellung ermittelt.

**Ergebnisse:**

Im Beobachtungszeitraum von 02/2016 bis 12/2020 wurden 458 Patient*inn*en (209 männlich, 249 weiblich, Durchschnittsalter 73,2 ± 9,9 Jahre) mit SCD (*n* = 44), naMCI (*n* = 40), aMCI (*n* = 182), AD (*n* = 120), MD (*n* = 68) und VD (*n* = 4) analysiert. Bei 109 Patient*inn*en (23,8 %) lagen die MR-Kriterien einer möglichen oder wahrscheinlichen CAA vor. Die CAA-Prävalenz war am höchsten bei Patient*inn*en mit aMCI (39,4 %) und MD (28,4 %). Bei 30,3 % der Patient*inn*en mit möglicher oder wahrscheinlicher CAA bestand eine Thrombozytenaggregationshemmung, bei 12,8 % eine DOAK-Therapie und bei 3,7 % eine Therapie mit Vitamin-K-Antagonisten. Die Anzahl kortikaler und subkortikaler Mikroblutungen war insgesamt bei Patient*inn*en mit gerinnungshemmender Therapie höher als bei Patient*inn*en ohne Gerinnungshemmung (*p* = 0,047). Ein Zusammenhang zwischen gerinnungshemmender Therapie und Häufigkeit kortikaler superfizieller Siderosen bildete sich nicht ab (*p* = 0,634).

**Diskussion:**

Die CAA ist bei Patient*inn*en mit kognitiven Störungen häufig. Bei nahezu der Hälfte der Patient*inn*en mit CAA besteht eine gerinnungshemmende Medikation. Eine gerinnungshemmende Medikation ist mit einer größeren Anzahl kortikaler und subkortikaler Mikroblutungen vergesellschaftet.

## Abstract (en)

**Objectives:**

To investigate the prevalence of coincident anticoagulation in patients with cognitive disorders and possible or probable cerebral amyloid angiopathy (CAA) as well as the relationship between the presence of oral anticoagulation and CAA-specific lesion load.

**Materials and methods:**

Patients with subjective cognitive decline (SCD), amnestic and non-amnestic mild cognitive impairment (aMCI/naMCI), Alzheimer’s disease (AD), mixed dementia (MD) and vascular dementia (VD) who presented to our outpatient dementia clinic between February 2016 and October 2020 were included in this retrospective analysis. Patients underwent cranial magnetic resonance imaging (MRI). MRI data sets were analyzed regarding the presence of CAA-related MRI biomarkers to determine CAA prevalence. Presence of anticoagulant therapy was determined by chart review.

**Results:**

Within the study period, 458 patients (209 male, 249 female, mean age 73.2 ± 9.9 years) with SCD (*n* = 44), naMCI (*n* = 40), aMCI (*n* = 182), AD (*n* = 120), MD (*n* = 68) and VD (*n* = 4) were analyzed. A total of 109 patients (23.8%) were diagnosed with possible or probable CAA. CAA prevalence was highest in aMCI (39.4%) and MD (28.4%). Of patients with possible or probable CAA, 30.3% were under platelet aggregation inhibition, 12.8% were treated with novel oral anticoagulants and 3.7% received phenprocoumon treatment. Regarding the whole study cohort, patients under oral anticoagulation showed more cerebral microbleeds (*p* = 0.047). There was no relationship between oral anticoagulation therapy and the frequency of cortical superficial siderosis (*p* = 0.634).

**Conclusion:**

CAA is a frequent phenomenon in older patients with cognitive disorders. Almost half of CAA patients receive anticoagulant therapy. Oral anticoagulation is associated with a higher number of cortical and subcortical microbleeds.

## Hintergrund und Fragestellung

Eine zerebrale Amyloidangiopathie (CAA) ist im höheren Lebensalter bei 20–40 % der Bevölkerung, bei Patient*inn*en mit Demenzerkrankungen sogar bei etwa 50–60 % nachweisbar [1]. Manifestationsformen der CAA sind intrazerebrale Lobärhämatome, subkortikale und kortikale Mikroblutungen (CMB), fokale Subarachnoidalblutungen und eine kortikale superfizielle Siderose (cSS), welche ein Residdum einer vorangegangenen fokalen Subarachnoidalblutng darstellt [2]. Neben dem Vorliegen einer disseminierten cSS und einer stattgehabten intrazerebralen Blutung (ICB) sind die Dauer und Art einer oralen Antikoagulation bedeutsame Prädiktoren für die Entwicklung einer ICB bei Patient*inn*en mit einer CAA [2]. Die orale Antikoagulation bei Patient*inn*en mit CAA stellt daher eine besondere therapeutische Herausforderung dar [2]. Die Assoziation der CAA zu Lobärblutungen, eine hohe ICB-Mortalität bei CAA und die hohe ICB-Rezidivneigung erfordern eine strenge und interdisziplinäre Risiko-Nutzen-Abwägung [2–5], was sowohl für die Behandlung mit Thrombozytenaggregationshemmern als auch für direkte orale Antikoagulanzien (DOAK) und für die Vitamin-K-Antagonisten Phenprocoumon und Warfarin gilt [2].

ASS erhöht in multivariaten Analysen das Risiko für eine Rezidiv-ICB bei Patient*inn*en mit CAA (HR 3,95, 95 %-KI 1,6–8,3; *p* = 0,021; [6]). Darüber hinaus ist die Therapie mit Thrombozytenaggregationshemmern bei Patient*inn*en > 60 Jahre mit einer höheren Prävalenz von CMB assoziiert [7]. 74 von 6045 Patient*inn*en (1,22 %) > 55 Jahre mit Vorhofflimmern entwickelten nach einer durchschnittlichen Behandlungsdauer mit einem oralen Antikoagulans oder einer Thrombozytenaggregationshemmung von 6 Jahren eine nichttraumatische ICB, wovon 51,4 % die diagnostischen Kriterien einer möglichen oder wahrscheinlichen CAA erfüllten [8]. Dabei waren keine Mortalitätsunterschiede zwischen der Thrombozytenaggregations- und der oralen Antikoagulationsgruppe feststellbar [8]. Die orale Antikoagulation mit Phenprocoumon bzw. Warfarin bei Patient*inn*en mit CAA erhöht das ICB-Risiko und die ICB-assoziierte Mortalität [9–11] und sollte daher unter Nutzen-Risiko-Abwägung indiziert werden. Bspw. kann bei speziellen Indikationen (z. B. mechanischer Aortenklappenersatz) die Indikation zu einer oralen Antikoagulationstherapie eher gestellt werden [2]. Das therapeutische Management bei einer Komorbidität von CAA und Vorhofflimmern ist aktuell unzureichend evidenzbasiert [12] und gleichzeitig von wachsender demografischer Bedeutung [3, 13]. Der interventionelle Vorhofohrverschluss stellt hier eine möglicherweise wirksame und hinsichtlich des ICB-Risikos sicherere therapeutische Alternative dar [12, 14, 15].

Neben dem Auftreten einer ICB kann die CAA auch in Form eines kognitiven Abbaus und transienter fokal-neurologischer Episoden (TFNE) in Erscheinung treten [16, 17]. TFNE sind klinisch schwer von transienten ischämischen Attacken (TIA) zu differenzieren [17], prädizieren jedoch ein hohes Rezidiv-ICB-Risiko, weshalb diese Differenzierung von hoher therapeutischer Relevanz ist [17]. Patient*inn*en mit zurückliegender ICB, mit kognitivem Abbau und TFNE sollten vor Beginn einer Thrombozytenaggregationshemmung oder oralen Antikoagulation daher eine MRT des Neurokraniums erhalten, um mögliche Korrelate einer CAA zu erkennen [2, 3].

Vor dem Hintergrund der hohen CAA-Prävalenz bei Patient*inn*en mit Demenzerkrankungen und dem erhöhten Blutungsrisiko unter gerinnungshemmender Medikation bei komorbider CAA untersucht diese retrospektive Analyse die Häufigkeit der CAA und einer gerinnungshemmenden Medikation in einer Kohorte von Patient*inn*en mit kognitiven Störungen. In einem zweiten Schritt werden explorativ Zusammenhänge zwischen dem Vorliegen einer gerinnungshemmenden Medikation und dem Vorhandensein bildmorphologischer CAA-Parameter analysiert.

## Studiendesign und Untersuchungsmethoden

### Studienteilnehmer

In dieser retrospektiven Analyse analysierten wir Patient*inn*en aus dem Dresdner Universitäts DemenzCentrum (UDC), die sich zwischen Februar 2016 und Dezember 2020 erstmals zur Demenzdiagnostik inklusive einer MRT des Neurokraniums mit Gradientenechosequenz [18] vorstellten. Eingeschlossen wurden konsekutive Patient*inn*en mit den Diagnosen einer subjektiven kognitiven Störung („subjective cognitive decline“ [SCD]; [19, 20]), eines nichtamnestischen MCI (naMCI), eines amnestischen MCI (aMCI; [21, 22]), einer Demenz vom Alzheimer-Typ (AD; [23, 24]), einer gemischten Demenz (MD) oder einer vaskulären Demenz (VD; [25]). Im Rahmen der Aktendurchsicht wurde ermittelt, ob und welche gerinnungshemmende Medikation zum Vorstellungszeitpunkt bestand. Die Patient*inn*en wurden dementsprechend kategorisiert: 1) keine gerinnungshemmende Medikation; 2) Thrombozytenaggregationshemmung oder 3) orale Antikoagulation (direkte orale Antikoagulanzien [DOAK] oder Vitamin-K-Antagonisten). Ein positives Ethikvotum der lokalen Ethikkommission lag vor (BO-EK-120022021).

### MRT-Evaluation und CAA-Diagnose

Die cMRT-Datensätze wurden durch eine erfahrene Neuroradiologin (JL) hinsichtlich des Vorliegens und des Ausmaßes CAA-spezifischer MRT-Biomarker analysiert. CMB wurden entsprechend ihrer Anzahl kategorisiert (0, 1, 2–4, 5–10, 10–50, > 50) und die cSS wurde unterteilt in abwesend oder fokal (< 4 Sulci). Die Diagnose einer möglichen bzw. wahrscheinlichen CAA wurde gemäß den modifizierten Boston-Kriterien gestellt [26–28]. Für die gegenständliche Analyse wurden aus der Gesamtkohorte nur die Probanden berücksichtigt, für die Angaben zu einer gerinnungshemmenden Medikation vorlagen. Eine Datenanalyse der CAA-Prävalenz und des CAA-Einflusses auf kognitive Parameter in der gesamten Studienkohorte unabhängig von einer gerinnungshemmenden Medikation befindet sich aktuell (Stand 31.07.2023) anderweitig unter Review.

### Statistische Auswertung

Demografische und klinische Charakteristika werden mittels Mittelwerten und Standardabweichungen (SD) für kontinuierliche Variablen und mittels absoluter und relativer Häufigkeiten für kategoriale Variablen beschrieben. In Abhängigkeit von Datenniveau und -verteilung wurden t‑Tests, Chi-Quadrat-Tests und ANOVAs durchgeführt. Die Zusammenhänge zwischen gerinnungshemmender Medikation und Anzahl von CMB sowie Ausprägungsgrad der cSS wurden mittels Chi-Quadrat-Test analysiert. Die statistische Auswertung erfolgte mit dem Programm IBM SPSS Statistics für Windows, Version 28.

## Ergebnisse

Im Beobachtungszeitraum wurden 458 konsekutive Patient*inn*en mit den oben genannten kognitiven Diagnosen (209 männlich, 249 weiblich) in die Analyse eingeschlossen, die eine cMRT mit Gradientenechosequenz erhalten haben. Das Durchschnittsalter betrug 73,2 ± 9,9 Jahre, 44 Patient*inn*en (9,6 %) erhielten die Diagnose einer SCD, bei 40 Patient*inn*en (8,7 %) wurde ein naMCI und bei 182 (39,7 %) ein aMCI diagnostiziert. 120 Patient*inn*en (26,2 %) erhielten die Diagnose einer AD und bei 68 (14,8 %) wurde eine MD diagnostiziert. Vier Patient*inn*en (0,9 %) erhielten die Diagnose einer VD (Tab. 1).

**Table 1:** 

		CAA						
	Alle	Keine	Möglich	Wahrscheinlich	*p*	CAA möglich oder wahrscheinlich	*p*	
*Anzahl, n*	*n* = 458	*n* = 349	*n* = 40	*n* = 69	–	*n* = 109	–	
*Durchschnittsalter (SD)*	73,2 (9,9)	71,9 (10,5)	76,5 (5,7)	77,6 (6,7)	< 0,001	77,2 (6,4)	< 0,001	ANOVA/t-Test
*Geschlecht (m)*	209 (45,6 %)	152 (43,6 %)	23 (57,5 %)	34 (49,3 %)	0,197	57 (52,3 %)	0,110	Chi^2^-Test
*Diagnose*								
SCD	44 (9,6 %)	37 (10,6 %)	5 (12,5 %)	2 (2,9 %)	–	7 (6,4 %)	–	
aMCI	182 (39,7 %)	139 (39,8 %)	22 (55,0 %)	21 (30,4 %)	–	43 (39,4 %)	–	
naMCI	40 (8,7 %)	35 (10,0 %)	2 (5,0 %)	3 (4,3 %)	–	5 (4,6 %)	–	
„Early onset AD“	9 (2,0 %)	7 (2,0 %)	0 (0 %)	2 (2,9 %)	–	2 (1,8 %)	–	
„Late onset AD“	111 (24,2 %)	91 (26,1 %)	4 (10,0 %)	16 (23,2 %)	–	20 (18,3 %)	–	
MD	68 (14,8 %)	37 (10,6 %)	7 (17,5 %)	24 (34,8 %)	–	31 (28,4 %)	–	
VD	4 (0,9 %)	3 (0,9 %)	0 (0 %)	1 (1,4 %)	< 0,001	1 (0,9 %)	< 0,001	Chi^2^-Test

*CAA* zerebrale Amyloidangiopathie, *SCD* subjektive kognitive Störung, *(n)aMCI* (nicht-)amnestisches „mild cognitive impairment“, *AD* Alzheimer-Demenz, *MD* gemischte Demenz, *VD* vaskuläre Demenz

Insgesamt wurde bei 109 Patient*inn*en (23,8 %) eine mögliche oder wahrscheinliche CAA nachgewiesen (mögliche CAA bei 40 Patient*inn*en [8,7 %]; wahrscheinliche CAA bei 69 Patient*inn*en [15,1 %]), wobei Patient*inn*en mit möglicher und wahrscheinlicher CAA älter waren als diejenigen ohne CAA (*p* < 0,001). Besonders hohe Prävalenzen einer möglichen und wahrscheinlichen CAA wurden bei Patient*inn*en mit aMCI (39,4 %) sowie bei Patient*inn*en mit gemischter Demenz (28,4 %) ermittelt, wobei die CAA-Prävalenz über die diagnostischen Gruppen hinweg inhomogen verteilt war (*p* < 0,001).

Von den 458 Patient*inn*en bestand insgesamt bei 124 (27,1 %) eine Thrombozytenaggregationshemmung (davon bei 2 Patient*inn*en eine duale Thrombozytenaggregationshemmung), bei 46 (10,0 %) eine Antikoagulation mit einem DOAK und bei 11 (2,4 %) eine Antikoagulation mit einem Vitamin-K-Antagonisten (alle mit Phenprocoumon). Von den 109 Patient*inn*en mit möglicher oder wahrscheinlicher CAA hatten 30,3 % eine Thrombozytenaggregationshemmung, 12,8 % eine orale Antikoagulation mit einem DOAK und 3,7 % waren mit einem Vitamin-K-Antagonisten (Phenprocoumon) antikoaguliert. Insgesamt bestand bei 46,8 % aller Patient*inn*en mit möglicher oder wahrscheinlicher CAA eine gerinnungshemmende Therapie (siehe Tab. 1 und 2).

**Table 2:** 

	CAA						
	Keine (*n* = 349)	Möglich (*n* = 40)	Wahrscheinlich (*n* = 69)	Möglich oder wahrscheinlich (*n* = 109)			
**Antikoagulation**							
Keine (*n* = 277)	219 (62,8 %)	21 (52,5 %)	37 (53,6 %)	58 (53,2 %)	–	Chi^2^	*P*
Thrombozytenaggregationshemmung (*n* = 124)	91 (26,1 %)	15 (37,5 %)	18 (26,1 %)	33 (30,3 %)	Keine vs. möglich + wahrscheinl.	3,866	0,276
DOAK (*n* = 46)	32 (9,2 %)	3 (7,5 %)	11 (15,9 %)	14 (12,8 %)	Keine vs. möglich	2,484	0,478
Vit.-K-Antagonist (*n* = 11)	7 (2,0 %)	1 (2,5 %)	3 (4,3 %)	4 (3,7 %)	Keine vs. wahrscheinl.	4,674	0,197

Die Anzahl kortikaler und subkortikaler Mikroblutungen war bei Patient*inn*en unter gerinnungshemmender Medikation höher als bei Patient*inn*en ohne Gerinnungshemmung, aber insgesamt war die Anzahl an Mikroblutungen pro Patient*in* in der gesamten Kohorte gering (siehe Tab. 3). Während die Häufigkeit kortikaler und subkortikaler Mikroblutungen in der Gruppe ohne gerinnungshemmende Medikation bei 20,9 % lag, fand sich die größte Häufigkeit unter Einnahme von DOAK (37,0 %), gefolgt von der Vitamin-K-Antagonisten-Gruppe (27,3 %) und der Gruppe unter Thrombozytenaggregationshemmung (26,6 %). Es bestand eine Assoziation zwischen dem Vorliegen einer gerinnungshemmenden Medikation (Thrombozytenaggregationshemmung, DOAK oder Vitamin-K-Antagonist) und der Anzahl kortikaler und subkortikaler Mikroblutungen (Tab. 3; *p* = 0,047).

**Table 3:** 

	CMB kortikal subkortikal					
	Keine	1	2–4	5–10	10–50	
**Antikoagulation**						
Keine (*n* = 277)	219 (79,1 %)	23 (8,3 %)	18 (6,5 %)	11 (4,0 %)	6 (2,2 %)	–
Thrombozytenaggregationshemmung (*n* = 124)	91 (73,4 %)	16 (12,9 %)	14 (11,3 %)	3 (2,4 %)	0 (0,0 %)	–
DOAK (*n* = 46)	29 (63,0 %)	3 (6,5 %)	7 (15,2 %)	5 (10,9 %)	2 (4,3 %)	**Chi-Quadrat-Test**
Vit.-K-Antagonist (*n* = 11)	8 (72,7 %)	0 (0,0 %)	2 (18,2 %)	1 (9,1 %)	0 (0,0 %)	*p* = 0,047

Hinsichtlich des Zusammenhangs zwischen dem Vorhandensein einer gerinnungshemmenden Therapie und der cSS-Last fand sich keine erhöhte cSS-Häufigkeit unter gerinnungshemmender Medikation (*p* = 0,634; siehe Tab. 4). Eine disseminierte cSS fand sich in der gesamten Kohorte nicht. Die Häufigkeit einer fokalen cSS war bei Patient*inn*en ohne gerinnungshemmende Therapie (2,9 %) ähnlich hoch wie bei Patient*inn*en unter Thrombozytenaggregationshemmung (2,4 %) und DOAK (2,2 %). Die größte Häufigkeit bestand bei Patient*inn*en unter Vitamin-K-Antagonist (9,1 %; siehe Tab. 4).

**Table 4:** 

	cSS		
	Keine	Fokal < 4 Sulci	
**Antikoagulation**			
Keine (*n* = 277)	269 (97,1 %)	8 (2,9 %)	–
Thrombozytenaggregationshemmung (*n* = 124)	121 (97,6 %)	3 (2,4 %)	–
DOAK (*n* = 46)	45 (97,8 %)	1 (2,2 %)	**Chi-Quadrat-Test**
Vit.-K-Antagonist (*n* = 11)	10 (90,9 %)	1 (9,1 %)	*p* = 0,634

## Diskussion

Diese retrospektive Datenanalyse verdeutlicht die hohe Prävalenz einer möglichen und wahrscheinlichen CAA bei Patient*inn*en mit kognitiven Störungen. Insbesondere bei Patient*inn*en mit den Diagnosen eines aMCI (39,4 %), einer MD (28,4 %) und einer AD (18,4 %), bei denen im Gegensatz zu den Diagnosen SCD, naMCI und VD am ehesten eine Alzheimer-Pathologie anzunehmen ist, war die Prävalenz einer komorbiden CAA hoch. Auch wenn die Boston-Kriterien nur für Patienten mit ICB validiert sind, bedeutet dies in der klinischen Praxis, dass das Vorliegen einer CAA bei älteren Patient*inn*en mit kognitiven Störungen in Betracht gezogen und die Durchführung einer kraniellen MRT mit Gradientenechosequenz in derartigen klinischen Konstellationen erwogen werden sollte. Darüber hinaus illustrieren die Daten die Häufigkeit einer gerinnungshemmenden Medikation bei Patient*inn*en mit einer möglichen oder wahrscheinlichen CAA. So bestand in der untersuchten Kohorte bei 30,3 % der Patient*inn*en mit bildgebenden Kriterien einer CAA eine Thrombozytenaggregationshemmung, bei 12,8 % eine Behandlung mit einem DOAK und bei 3,7 % eine Vitamin-K-Antagonisten-Therapie.

Aktuelle Daten zeigen, dass das ICB-Risiko bei Patient*inn*en mit zerebralen Mikroblutungen per se und unabhängig von einer oralen Antikoagulation erhöht ist und dass dieses Risiko von der Anzahl der Mikroblutungen abhängt [29]. Vor diesem Hintergrund sollte bei der Indikationsstellung für eine orale Antikoagulation bei CAA das Basisrisiko für ICB durch den Ausprägungsgrad CAA-spezifischer Läsionen Berücksichtigung finden.

In unserer Kohorte wiesen Patient*inn*en unter oraler Antikoagulation insgesamt mehr kortikale und subkortikale Mikroblutungen auf als Patient*inn*en ohne orale Antikoagulation, wobei die Anzahl der Mikroblutungen pro Patient*in *in der untersuchten Kohorte insgesamt gering war. Ein Zusammenhang zwischen dem Vorliegen einer gerinnungshemmenden Therapie und einer größeren Häufigkeit einer cSS ließ sich nicht nachweisen, wobei eine fokale cSS mit 9,1 % am häufigsten in der Vitamin-K-Antagonisten-Gruppe nachweisbar waren. Dass insgesamt nicht ein einziger Fall einer disseminierten Siderose auftrat, ist wahrscheinlich der insgesamt doch limitierten Fallzahl geschuldet. Möglicherweise besteht aber auch ein Bias dadurch, dass es sich bei den Probanden primär um Patienten mit kognitiven Störungen handelte, die sich möglicherweise in einem noch frühen CAA-Erkrankungsstadium befanden.

Eine CAA wird in einem beträchtlichen Anteil nichttraumatischer ICB-Fälle als ursächlich angenommen [2]. Zudem sind spontane ICB bei bestehender CAA mit einer hohen Mortalität assoziiert [2]. Dauer und Art der oralen Antikoagulation stellen dabei bedeutsame Prädiktoren für die Entwicklung einer ICB bei bestehender CAA dar [2]. Insbesondere die Behandlung mit Vitamin-K-Antagonisten erhöht das ICB-Risiko bei Patient*inn*en mit CAA um den Faktor 7–10 und die ICB-assoziierte Mortalität um 60 % [9–11]. Die aktuell begrenzte Datenlage lässt auch eine Risikoerhöhung für ICB bei vorliegender CAA unter Therapie mit DOAK sowie unter Thrombozytenaggregationshemmung vermuten [2]. Vor diesem Hintergrund sensibilisieren die vorliegenden Daten für die demografisch bedeutsamer werdende Koinzidenz einer oralen Antikoagulation und einer CAA, insbesondere bei Patientinnen mit begleitenden kognitiven Störungen. In Anbetracht bestehender therapeutischer Alternativen zur oralen Antikoagulation in bestimmten klinischen Situationen, beispielsweise in Form eines interventionellen Vorhofohrverschlusses, sind diese Daten wichtig, um Behandler auf diese Koinzidenz aufmerksam zu machen, damit diese therapeutischen Alternativen in entsprechenden Situationen geprüft bzw. in Studien weiter evaluiert werden. Bisherige Studienergebnisse legen nahe, dass der Vorhofohrverschluss eine effektive und sichere Alternative zur Antikoagulation darstellt [30]. Anzumerken ist jedoch, dass Patienten mit hohem Blutungsrisiko in den ersten Studien ausgeschlossen wurden und dass aktuell keine einheitlichen Empfehlungen bezüglich der postinterventionellen Thrombozytenaggregationshemmung existieren [30].

Patient*inn*en mit einem erhöhten kardiovaskulären Risiko erhalten häufiger eine gerinnungshemmende Therapie, weisen aber gleichzeitig ein erhöhtes Risiko für zerebrale Mikroblutungen auf [7]. Neben diesem „confounding-by-indication bias“ limitieren ein häufig retrospektives Design ähnlicher Untersuchungen und eine zeitlich unzureichend definierte Hämosiderinnachweisdauer in möglicherweise vor Therapiebeginn vorbestehenden zerebralen Mikroblutungen Aussagen zu Kausalzusammenhängen zwischen oraler Antikoagulation und CAA-spezifischer Läsionslast [2, 7].

Neben diesen auch in unserer Analyse gegenständlichen Limitationen ist außerdem darauf zu verweisen, dass in der untersuchten Kohorte weder die Indikation für die jeweilige gerinnungshemmende Therapie noch die vorbestehende Dauer der oralen Antikoagulation miterfasst wurde, was die Aussagen zu Zusammenhängen zwischen dem Vorliegen einer gerinnungshemmenden Therapie und der Häufigkeit von kortikalen und subkortikalen Mikroblutungen bzw. cSS relativiert. Ferner begrenzen fehlende Informationen zum bildgebenden Verlauf, wie z. B. zu vorbestehenden Mikroblutungen, und somit zum tatsächlichen Blutungsrisiko im Verlauf Aussagen zu diesen Zusammenhängen. In Übereinstimmung mit anderen Daten lässt diese Untersuchung jedoch vermuten, dass eine orale Antikoagulation mit häufigeren kortikalen und subkortikalen Mikroblutungen vergesellschaftet ist, was sich in der untersuchten Kohorte für cSS nicht nachweisen ließ.

Diese Analyse von *Real-life*-Daten aus der klinischen Versorgungsrealität beschreibt somit primär die Häufigkeit der CAA bei älteren Patient*inn*en mit kognitiven Störungen sowie die Häufigkeit der Koinzidenz von CAA und gerinnungshemmender Therapie.

## Fazit für die Praxis

- Die CAA ist insbesondere bei älteren Patient*inn*en mit kognitiven Störungen häufig und sollte vor Beginn einer gerinnungshemmenden Therapie in Betracht gezogen werden.
- Bei älteren Patient*inn*en mit kognitiven Störungen sollte die Durchführung einer MRT mit hämsensitiven, suszeptibilitätsgewichteten MRT-Sequenzen (SWI, GRE etc.) erwogen werden, um eine evtl. vorliegende CAA zu detektieren.
- Eine gerinnungshemmende Therapie ist in der klinischen Praxis bei Patient*inn*en mit bildgebenden Kriterien einer CAA häufig.
- Daten zu Kausalzusammenhängen zwischen dem Vorliegen einer oralen Antikoagulation und der CAA-spezifischen Blutungslast sind zum aktuellen Zeitpunkt aus methodischen Gründen begrenzt.
